# Improving the Performance of Pseudo-Random Single-Photon Counting Ranging Lidar

**DOI:** 10.3390/s19163620

**Published:** 2019-08-20

**Authors:** Yang Yu, Bo Liu, Zhen Chen

**Affiliations:** 1 Institute of Optics and Electronics, Chinese Academy of Sciences, Chengdu 610209, China; 2 College of Materials Science and Opto-Electronic Technology, University of Chinese Academy of Sciences, Beijing 100049, China

**Keywords:** Lidar, photon counting, detection, modulation

## Abstract

A new encoding method is proposed to improve the performance of pseudo-random single-photon counting ranging (PSPCR) Lidar. The encoding principle and methodology are presented. In addition, the influence of detector’s dead time on the detection probability is analyzed with theoretical derivation and Monte Carlo simulation. Meanwhile, we propose using macro code as the analysis unit to quantitatively analyze the detection probability and single-photon detection efficiency of the traditional PSPCR Lidar and the modulated PSPCR Lidar. The Monte Carlo simulation and experiment prove that the proposed method exhibited better ranging performance than the traditional PSPCR Lidar system.

## 1. Introduction

In recent years, there has been increasing interest in the research on photon counting Lidar for three-dimension imaging [1], topographic measurements from airborne and spaceborne platform [2], atmospheric sensing [3]. Due to single-photon events detection capability, Geiger-mode avalanche photodiode (GM-APD) detectors have been gaining popularity and are used for the long-range or weak signal detection. However, the background-light and dark counting will prevent the detector from reaching the desired detection ability. Accumulating multiple pulses technique was proposed to improve the detection probability and signal-to-noise ratio by several researchers [4,5,6]. However, the accumulation numbers of pulses will affect the speed and efficiency of detection, which makes the Lidar unsuitable for the detection of high-dynamic targets. McCarthy [7] utilized a high repetition rate laser source (tens of MHz) to reduce the data acquisition time. However, the high repetition rate would greatly reduce the maximal unambiguous distance. Then, using a finite non-periodic pulse train or pulse-position modulation technique to resolve range ambiguity was demonstrated by several researchers [8,9,10]. In 1983, Takeuchi et al. first proposed using a random phase code to modulate continuous-wave laser with an external optical modulator, as a transmitting laser source in laser ranging system [11]. Gradually, pseudo-random coding ranging system has become a research hotspot [12,13,14]. 

Though the GM-APD is widely used for the long-range or weak signal detection, its efficiency is significantly reduced by the inability to detect more than one photon per reset due to its dead time [5], which causes a nonlinear effect. For this, the article analyzes the influence of the dead time on the pseudo-random photon-counting detection probability and puts forward a solution based on the theory of pseudo-random coding ranging and single-photon detection. 

## 2. The Theoretical Analysis

In this part, the traditional pseudo-random coding ranging basic theory is described and the pseudo-random single-photon counting ranging (PSPCR) detection probability is analyzed. 

### 2.1. Pseudo-Random Ranging Theory

**Figure 1:**
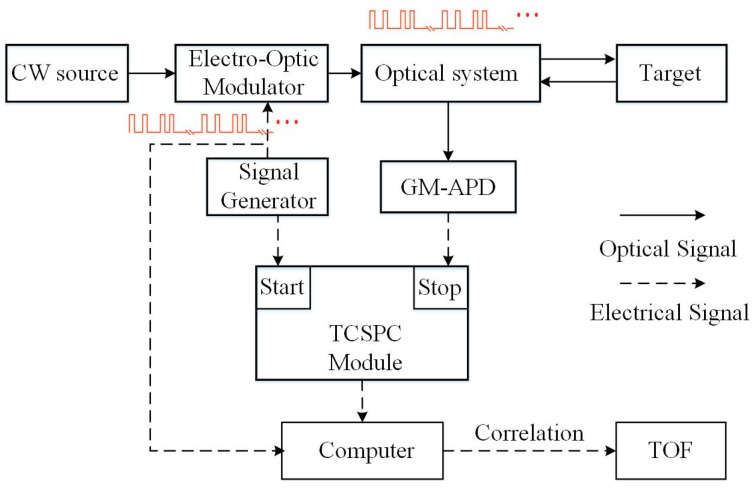
Schematic of pseudo-random ranging system.

The pseudo-random ranging method is a time-resolved measurement technique, which is derived from the spread spectrum time-resolved optical measurement method [15]. For the PSPCR technique, the emitted pulses are modulated to pseudo-random code sequences, while the echo sequences are detected by the single-photon detector. A cross-correlation function is generated from the detected echo signal and the transmitted pseudo-random sequence. The impulse response extracted from the auto-correlation function yields the target’s distance. As shown in Figure 1, the schematic diagram of the pseudo-random modulation ranging system is given.

**Figure 2:**
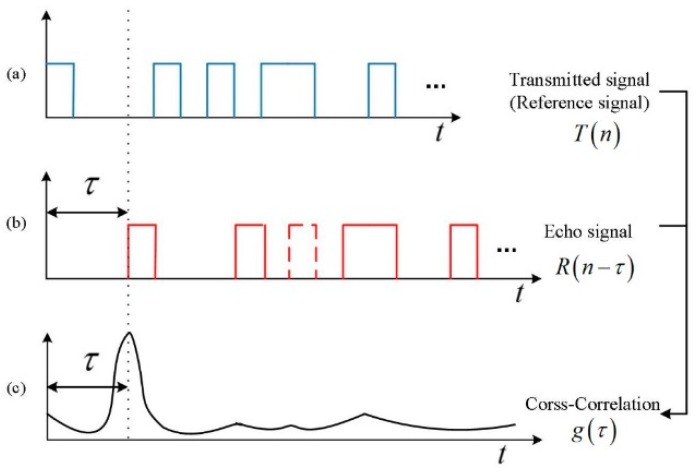
The schematic diagram of pseudo-random ranging principle. (**a**) The transmitted pseudo-random laser pulse sequence (reference signal); (**b**) The detected pulse sequence of the target response; (**c**) The auto-correlation function of the reference and the target response.

As shown in Figure 1, the electro-optic modulator modulates the continuous laser into a pseudo-random pulse sequence. The modulation signal of electro-optic modulator is a traditional pseudo-random sequence, or a modulation-encoded pseudo-random sequence generated by the signal generator. The pseudo-random sequences are periodically emitted. Each cycle contains only a set of pseudo-random sequence. The longer the period is, the longer the unambiguity distance of the system is. Generally, the length of the period is much longer than that of the pseudo-random sequence. As shown in Figure 2, the T(n) is the transmitted signal of N-bit long pseudo-random sequence, and R(n) is the received echo pseudo-random sequence, where the dashed pulse indicates an echo pulse that is not responded by the GM-APD. Mathematically, each element of the R(n) and T(n) sequence should be a bipolar sequence and have an absolute value of 1, and they have a periodic auto-correlation function similar to a delta function:

(1) $$g\left(\tau \right)=\frac{1}{N}\sum_{n=1}^{N}R\left(n-\tau \right)T\left(n\right)=\left\{ \begin{array}{ll} 1, & \tau =\pm 2R/c,\pm 2\cdot \left(2R/c\right),\cdot \cdot \cdot \\ \approx 0, & \tau \neq \pm 2R/c,\pm 2\cdot \left(2R/c\right),\cdot \cdot \cdot \end{array}\right.,$$

At a time delay τ, the auto-correlation function g(τ) has the maximum value, then the distance of the target can be calculated by the formula, R=cτ/2.

### 2.2. Dead Time Effects on Pseudo-Random Single Photon Ranging

For each bit, the detection probability is impacted by three aspects. First, the GM-APD shows nonlinearity due to the dead time, which is characterized by the probability that the device is armed and able to respond to up to one photon event, $P_{A}$. Second, the detection probability makes sense only when a pulsed laser is emitted in the pseudo-random sequence, T(n). Finally, even if the GM-APD is armed and the pulse is transmitted, the detection probability also takes into account the effect of the trigger probability of primary photoelectrons, $P_{S}$. Thus, for the PSPCR system, the detection probability, $P_{D}$, can be written as

(2) $$P_{D}\left(n\right)=P_{A}\left(n\right)\cdot P_{S}\cdot T\left(n\right),$$

When the number of primary photoelectrons caused by echo signal, background noise, and dark current is low, the detection model of the GM-APD follows Poisson statistics approximately [16]. For a Poisson process, the probability density function (PDF) describes the probability that k events occur in a counting interval. This trigger probability density function distribution is given by

(3) $$P_{S}\left(k\right)=\frac{1}{k!}{\left(M\right)}^{k}\exp\left(-M\right),$$

where $M=M_{s}+M_{n}$. $M_{s}$ is the mean of echo signal primary photoelectron [15] numbers, and $M_{n}$ is the mean of noise photoelectron numbers, which includes two components, background noise, and dark counting noise. k is the number of primary photoelectrons. As shown in Equation (3), the probability of no primary photoelectrons is exp(−M). The GM-APD is triggered when at least one event occurs. The trigger probability is

(4) $$P_{S}\left(k>0\right)=1-\exp\left(-M\right),$$

In practice, the GM-APD always has a dead time of tens of nanoseconds to even microsecond [17,18]. It cannot respond to other photon events during the dead time. As the bit modulating rate is usually GHz in the PSPCR method, the bit width is much smaller than the dead time of common detectors. Thus, the following bits may be affected by the previous bits into the detector’s dead zone, cannot be responded by the detector. As a result, the GM-APD arm probability varies greatly among different bits in pseudo-random sequence.

Considering the trigger probability $P_{S}$ and the impact of dead time, the GM-APD arm probability of N bits pseudo-random sequence is

(5) $$P_{A}\left(n\right)=\left\{ \begin{array}{ll} 1-\sum_{i=1}^{n-1}P_{S}\cdot P_{A}\left(i\right)\cdot T\left(i\right), & n\leq b\\ 1-\sum_{i=n-b}^{n-1}P_{S}\cdot P_{A}\left(i\right)\cdot T\left(i\right), & b<n\leq N \end{array}\right.,$$

where $b=\left\lceil t_{d}/\Delta t\right\rceil$ is the number of bits per dead time, where $t_{d}$ is the dead time and Δt is bit width.

By using the expression from Equation (5) in Equation (2), the detection probability $P_{D}$ of N bits pseudo-random sequence is

(6) $$\begin{array}{ll} P_{D}\left(n\right) & =P_{A}\left(n\right)\cdot P_{S}\cdot T\left(n\right)\\ & =\left\{ \begin{array}{ll} P_{S}\cdot (1-\sum_{i=1}^{n-1}P_{S}\cdot P_{A}\left(i\right)\cdot T\left(i\right))\cdot T\left(n\right), & n\leq b\\ P_{S}\cdot (1-\sum_{i=n-b}^{n-1}P_{S}\cdot P_{A}\left(i\right)\cdot T\left(i\right))\cdot T\left(n\right), & b<n\leq N \end{array}\right. \end{array}$$

**Figure 3:**
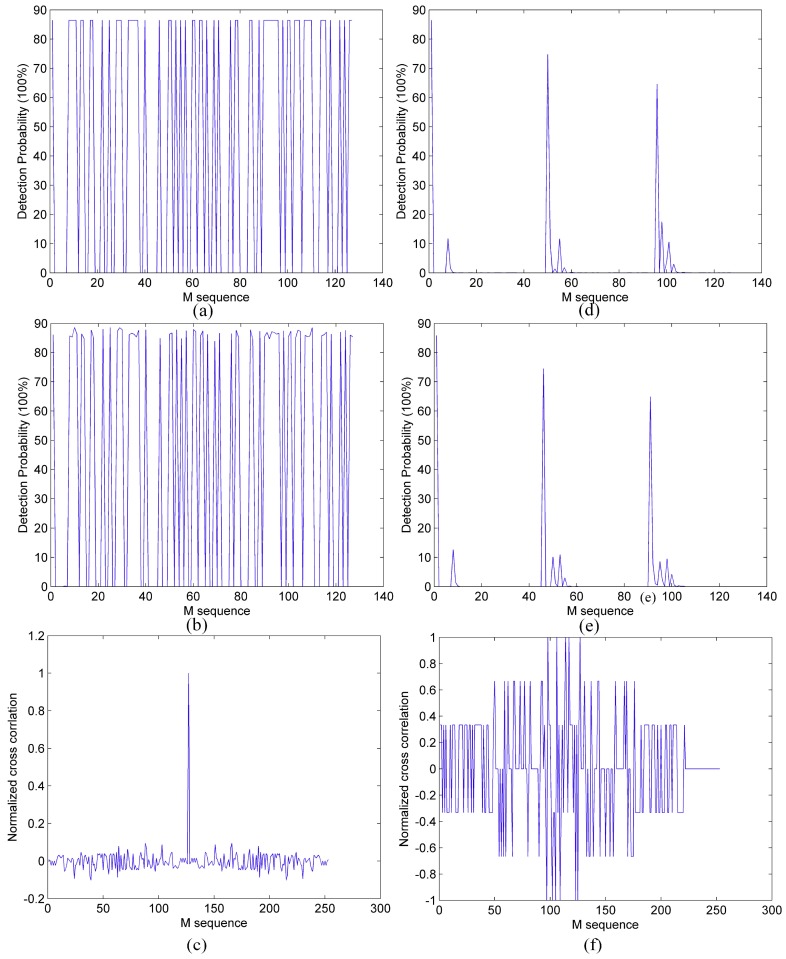
The detection probability of each code in pseudo-random single-photon counting ranging (PSPCR) Lidar system and the cross-correlation function. (**a**,**d**) are the PSPCR method detection probabilities of theory derivation while the dead time is 0 and 45 ns, respectively; (**b**,**e**) are the PSPCR method detection probabilities of Monte Carlo simulation while the dead time is 0 and 45 ns, respectively; (**c**,**f**) are the normalized cross-correlations of the PSPCR method.

In order to better analyze the influence of dead time on the detection probability of PSPCR Lidar, the numerical simulation of Equation (6) is conducted under two different dead time conditions (0 and 45 ns). In addition, the numerical simulation results are verified by Monte Carlo simulation. The parameters, which are used in these simulations are listed in Table 1. The simulation results are shown in Figure 3.

As can be seen from Figure 3a,b or Figure 3d,e, the Monte Carlo simulation result of the PSPCR method detection probability is in agreement with the theoretical derivation. In other words, it confirms the correctness of our theoretical derivation. In this simulation, we perform correlation operations with a periodic M-sequence and modulation-encoded M-sequence. Analyzing Figure 3a,d or Figure 3b,e, the dead time has two negative effects on the PSPCR method. First, the number of detected ‘1’ bits in the pseudo-random sequence is decreased. Second, average probabilities of all ‘1’ bits are reduced. Analyzing Figure 3d,e, the sum of detection probability of the pseudo-random sequence bits in per dead time is approximately 100%, because the detector responds to at most one photon event per dead time. What is more, as can be seen in Figure 3c, when the dead time is 0 ns, the maximum value of cross-correlation function is obvious. However, when the dead time is relatively long, the PSPCR method cannot be correctly ranging. In Section 3, using the modulated pseudo-random sequence to improve the performance of PSPCR method is introduced.

## 3. Improved Pseudo-Random Coding Single-Photon Detection Method

### 3.1. The Modulated Pseudo-Random Sequence

**Figure 4:**
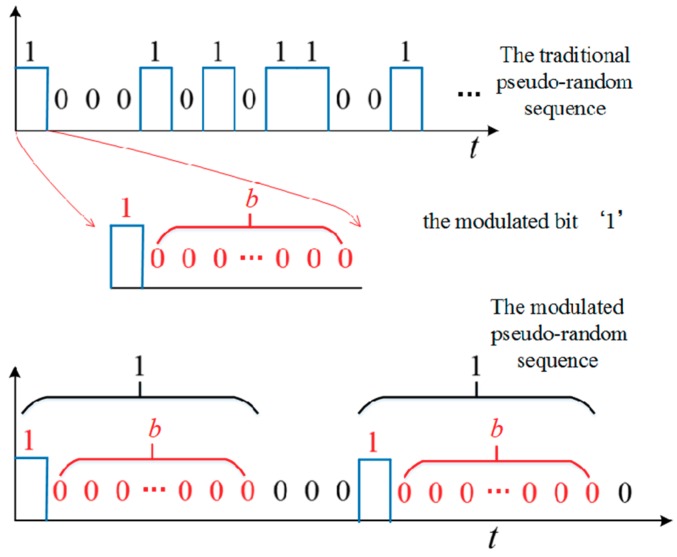
The schematic diagram of the modulated pseudo-random sequence.

The dead time has two negative effects on the PSPCR Lidar system. In particular, the current bit of pseudo-random sequence either steps into the dead zone or decreases the detection probability due to the effects of the front bits. To solve the problem, this article proposes a modulated pseudo-random encoding method. The traditional pseudo-random code sequences contain ‘0’ and ‘1’. They all represent one bit and have the same bit width, where ‘1’ represents an emitting pulse and ‘0’ represents no transmitting pulse. In order to reduce the negative effects of dead time mentioned in Section 2.2, we modulate the ‘1’ bits of the traditional pseudo-random sequences. The principle is shown in Figure 4.

As shown in Figure 4, the traditional ‘1’ bits are replaced by one real ‘1’ bit and b ‘0’ bits. Thus, the ‘1’ bits of the modulated pseudo-random sequence are independent of each other and are no longer affected by the other ‘1’ bits stepping into the dead zone.

### 3.2. Detection Performance Analysis

There are many ways to characterize the performance of Lidar system. Detection probability and single-photon detection efficiency (SPDE) are typical evaluation metrics. This paper chooses these two metrics to compare the performance of the traditional PSPCR Lidar and modulation-encoded PSPCR Lidar.

#### 3.2.1. Detection Probability

Equation (6) gives the detection probability of each bit in the pseudo-random sequence, but it is difficult to visually represent the detection probability of the PSPCR Lidar system. Thus, we propose a more obvious way to express the detection probability of the PSPCR Lidar system. In this method, the pseudo-random sequence is divided into multiple macro codes. The GM-APD responds to at most one bit within a dead time length, so we use the dead time length as the division unit of macro code. The detection probability of per macro code is affected by the number of echo photoelectrons, which can be written as

(7) $$\left\{ \begin{array}{l} P_{Dbin}=\sum_{i=1}^{d}P_{D}\left(i\right)\cdot \prod_{j=1}^{i-1}\left(1-P_{D}\left(j\right)\right)\\ \sum_{i=1}^{d}P_{D}\left(i\right)\leq 1 \end{array}\right.,$$

**Figure 5:**
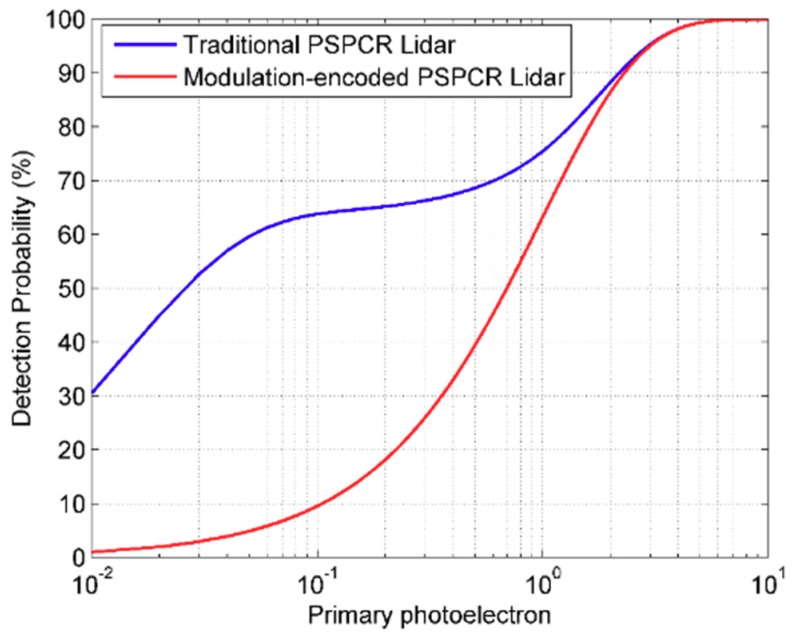
The detection probability of the traditional PSPCR Lidar and modulation-encoded PSPCR Lidar at different primary photoelectron number.

As shown in Figure 5, when the number of primary photoelectrons is small, the detection probability of the modulation-encoded PSPCR Lidar is lower than the detection probability of the PSPCR Lidar system. With the number of primary photoelectrons increasing, the gap gradually decreases. The main reason is that the PSPCR Lidar transmits more ‘1’ bit in a dead time period than the modulation-encoded PSPCR Lidar, and the detection probability increases by accumulating multiple ‘1’ bits. With the increase of the number of primary photoelectrons, the detection probability of a single ‘1’ bit gradually increases and gradually becomes saturated. 

However, it should be pointed out that the macro code detection probability of the traditional PSPCR Lidar is higher than that of the modulation-encoded PSPCR Lidar because it contains more ‘1’ bits in per macro code. However, at most one ‘1’ bit per macro code can be responded to by the GM-APD. Other ‘1’ bits that cannot be detected will become error bits and affect the ranging performance of the system. 

#### 3.2.2. Signal Photon Detection Efficiency

The SPDE, $\eta_{s}$, is the probability that a signal PE is detected, $P_{D}$, divided by the mean number of single photon $n_{s}$ incidents on the GM-APD. That is

(8) $$\eta_{s}=P_{D}/n_{s},$$

Because the GM-APD can only respond to at most one photon event in each dead time length, the SPDE of PSPCR Lidar and modulation-encoded PSPCR Lidar system are analyzed based on the dead time period (macro code). Then, Equation (8) can be reformulated to

(9) $$\eta_{s}=P_{Dbin}/n_{s}=\sum_{i=1}^{b}P_{D}\left(i\right)\cdot \prod_{j=1}^{i-1}\left(1-P_{D}\left(j\right)\right)/n_{s},$$

**Figure 6:**
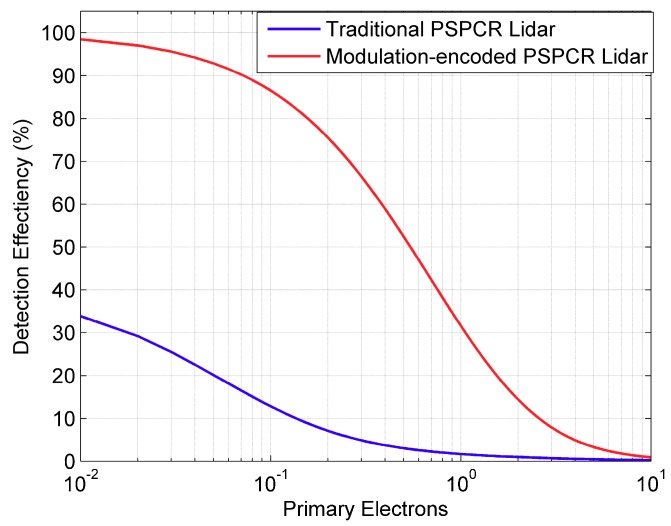
The signal photon detection efficiency of the traditional PSPCR Lidar and modulation-encoded PSPCR Lidar at different primary photoelectron number.

For the PSPCR Lidar system, the $n_{s}\approx \frac{b}{2}M_{s}$ is the primary photoelectrons number of a macro code in the traditional pseudo-random sequence. However, for modulation-encoded PSPCR Lidar, the $n_{s}\approx M_{s}$, because there is only one ‘1’ bit in a macro code, when the pseudo-random sequence is modulated. As shown in Figure 6, the SPDE of modulation-encoded PSPCR Lidar is always greater than that of the traditional PSPCR Lidar, especially when the number of primary photoelectrons is relatively small. As the traditional PSPCR Lidar transmits more than one ‘1’ bits in a macro code, at most one of these ‘1’ bits are detected, which makes traditional PSPCR Lidar have lower SPDE.

As can be seen in Figure 5 and Figure 6, the modulation-encoded PSPCR Lidar has higher SPDE than the traditional PSPCR Lidar, but the detection probability is lower than the traditional PSPCR Lidar. In the next section, the Monte Carlo simulations will be used to prove that although the modulation-encoded PSPCR Lidar has a lower detection probability than the traditional PSPCR Lidar, its ranging capability is still higher than that of the traditional PSPCR Lidar.

### 3.3. Monte Carlo Simulation

The Monte Carlo simulations of the traditional pseudo-random sequence and the modulation-encoded pseudo-random sequence are implemented. The parameters used in both simulations are listed in Table 1. To improve the traditional PSPCR ranging performance, the degree of M sequence in Table 1 is changed from 7 to 10, and the target distance is set to 300 meters.

**Figure 7:**
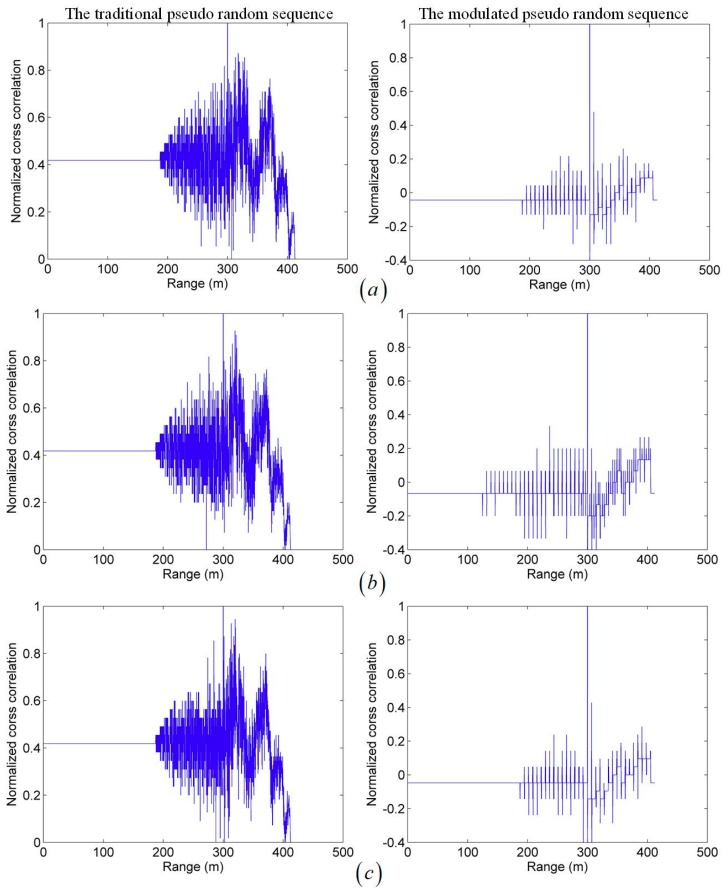
Cross-correlation range images with three different levels of noise photoelectrons. The first column is the Monte Carlo simulation of the traditional pseudo-random sequence, while the second is the modulated pseudo-random sequence. The noise levels of (**a**–**c**) are represented by the mean number of photoelectron noise. They are $1\times 10^{-4}$, $5\times 10^{-4}$ and $10\times 10^{-4}$ per bit, respectively.

Both simulations transmit the same length pseudo-random sequence with 1GHz bit rate. In the two simulations, three different levels of noise photoelectrons are set up. Since the noise and echo signal are subject to the Poisson distribution, their mean values are listed to represent the distribution characteristics. The simulations’ results are shown in Figure 7.

It can be seen in Figure 7 that for three different levels of noise photoelectrons both methods can measure the distance correctly. However, comparing the first column with the second of Figure 7, the modulation-encoded PSPCR Lidar can distinguish the target distance more clearly than the traditional PSPCR Lidar. In other words, the peak of cross-correlation function in the second method is more obvious. The traditional PSPCR Lidar increases the detection probability within a dead-time unit by transmitting more ‘1’ bits. However, it makes more bits undetectable, causing more error bits in cross-correlation operations. Thus, sidelobe noise of the cross-correlation function is high, and it is difficult to extract the peak position of the echo signal, which makes its ranging capability lower than that of modulation-encoded PSPCR Lidar. Moreover, with the increase of noise intensity, the performance of the traditional PSPCR Lidar is significantly worse than the modulation-encoded PSPCR Lidar. Therefore, the proposed method is better in terms of range performance and has more obvious anti-noise ability.

## 4. Experiment

**Figure 8:**
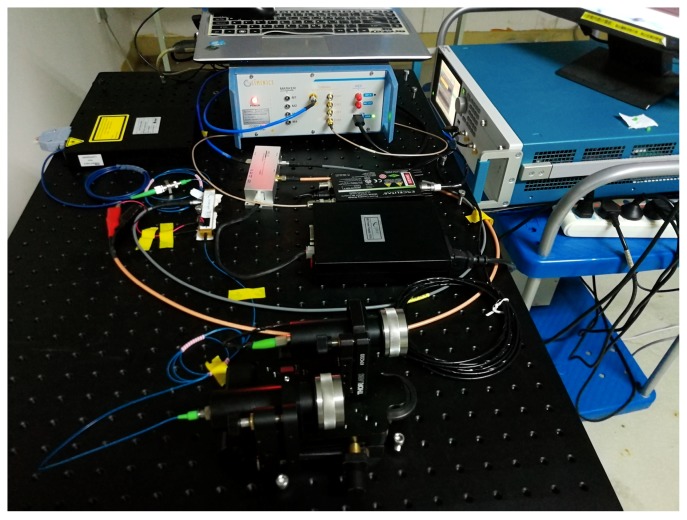
Experiment platform for the modulation-encoded PSPCR Lidar and the traditional PSPCR Lidar.

In order to prove the effectiveness of the proposed method, we built an experimental platform. GM-APD (SPCM-NIR-10-FC), signal generator (AGW5000) and electro-optic modulator (IM-1064-10-PM) were used in the experiment. The distance measurement of the carton at 10.5 m was completed. The performance of the modulation-encoded PSPCR Lidar and the traditional PSPCR Lidar under different echo signal intensities was compared. The experimental platform is shown in Figure 8. The main parameters of the two Lidar systems are given in Table 2.

**Figure 9:**
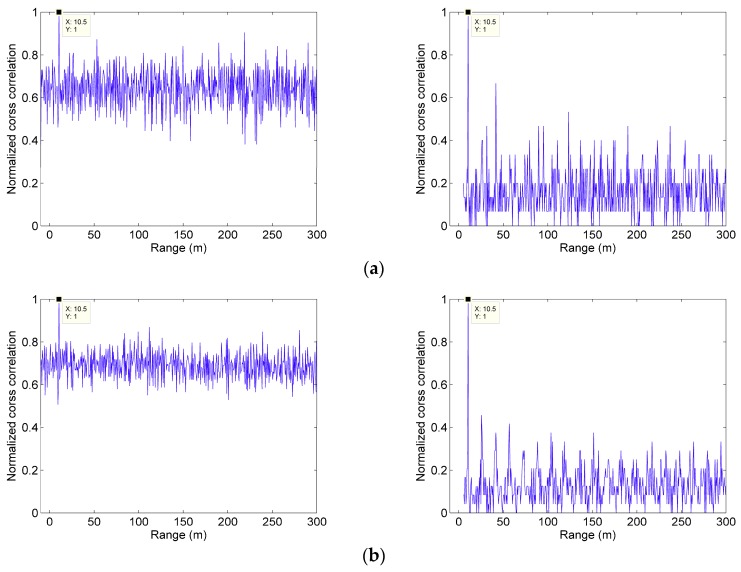

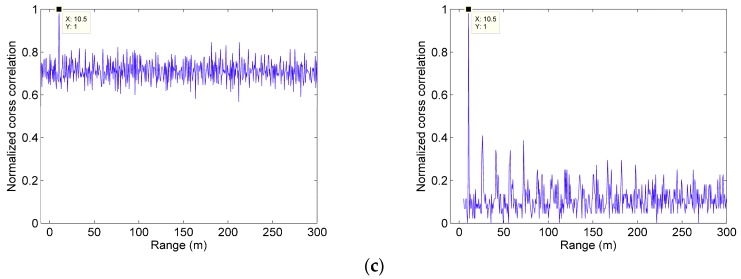
Cross-correlation range images with three different echo photon numbers for the traditional PSPCR Lidar and the modulation-encoded PSPCR Lidar. The first column is the traditional PSPCR Lidar, and the second column is the modulation-encoded PSPCR Lidar. The mean echo photon number per ‘1’ bit in (**a**), (**b**) and (**c**) is 1, 3, and 5, respectively.

When the noise count is 1 Mcps, we compare the cross-correlation function of the traditional PSPCR Lidar and the modulation-encoded PSPCR Lidar under different echo signal intensities. As shown in Figure 9, the first column is the cross-correlation functions of the traditional PSPCR Lidar and the second column is the modulation-encoded PSPCR Lidar. Figure 9a,b,c shows the cross-correlation functions of the two methods when the mean echo signal photon number of per ‘1’ bit is 1, 3, and 5, respectively. Under the same echo signal intensity, the side-lobe of the modulation-encoded PSPCR Lidar is always lower than that of the traditional PSPCR Lidar. Therefore, we can infer that the ranging performance of the modulation-encoded PSPCR Lidar is always better than that of the traditional PSPCR Lidar at the same echo signal intensity and noise level. 

**Figure 10:**
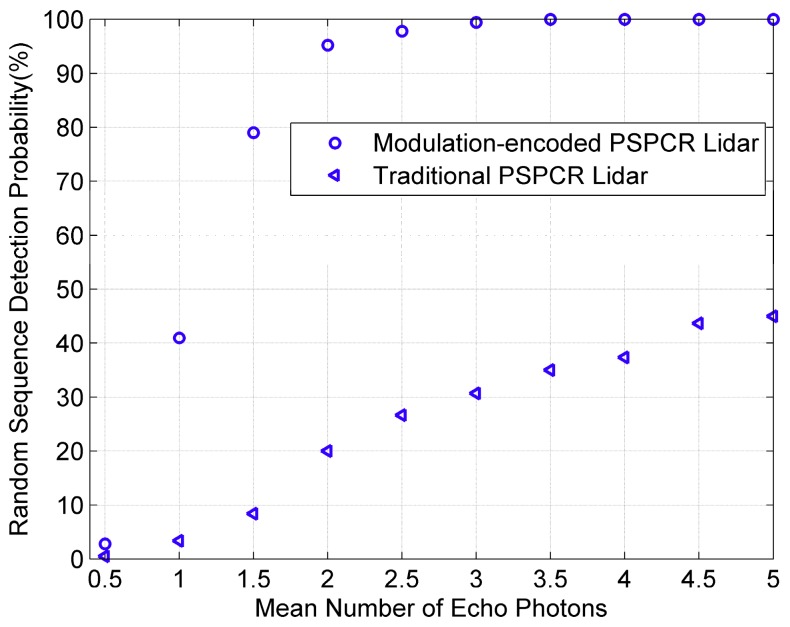
The detection probability statistical results of the modulation-encoded PSPCR Lidar and the traditional PSPCR Lidar at different mean echo photon number.

The probability that the Lidar can correctly identify targets is an important index to evaluate the ranging performance. When the noise counts are 1 Mcps, the number of echo photon is 0.5–5, 500 experiments are complete at each echo signal level, respectively. The probability of correctly detecting target at different echo signal levels is shown in Figure 10.

Figure 10 shows the change trend of target detection probability of the modulation-encoded PSPCR Lidar and the traditional PSPCR Lidar with the number of echo photons. It can be found that the detection probability of both methods increases with the increase of the mean number of echo photons. However, the detection probability of the modulation-encoded PSPCR Lidar is always significantly higher than that of the traditional PSPCR Lidar except when the mean number of echo photons is 0.5. And when the mean number of echo photons is three, the detection probability of the modulation-encoded PSPCR Lidar reaches saturation. The main reason is that, due to the influence of detector dead time, there are many ‘1’ bits in the traditional PSPCR Lidar which cannot be responded by the GM-APD because it enters the dead zone. Therefore, compared with the modulation-encoded PSPCR Lidar, the traditional PSPCR Lidar will produce more error bits, which makes its ranging performance worse than that of the modulation-encoded PSPCR Lidar. Experiments show that the proposed method can effectively improve the ranging performance of the PSPCR Lidar system.

## 5. Conclusions

In this paper, a modulation-encoded pseudo-random laser pulse sequence is used in the PSPCR Lidar system to replace the traditional pseudo-random laser pulse sequence, where a GM-APD is used to record the echo pulse sequence. Aiming at the problem that the traditional pseudo-random sequence is affected by the dead time, its detection probability of per bit is analyzed for the PSPCR Lidar. It is proved by both theory and Monte Carlo simulation that the detection probability of ‘1’ bits decreases when considering the effect of dead time on the traditional PSPCR Lidar. With the decrease of the detection probability of the ‘1’ bits, the correlation function of traditional pseudo-random sequences becomes worse, which leads to the poor performance of the traditional PSPCR Lidar. We propose to use a modulated pseudo-random sequence to reduce the negative effects of dead time, improving the correlation of pseudo-random sequences. The single photon detection efficiency of the improved PSPCR Lidar system is higher than that of the traditional PSPCR Lidar, but the detection probability is lower than that of the traditional PSPCR Lidar. However, Monte Carlo simulations and experiments have found that although the traditional PSPCR Lidar system has a relatively high detection probability, its peak correlation function is not as obvious as that of an improved PSPCR Lidar system due to more error bits. Therefore, the ranging performance of modulation-encoded PSPCR Lidar has a more obvious improvement than that of the traditional PSPCR Lidar.

## Figures and Tables

**Table 1:** Simulation parameters of pseudo-random single-photon detection.

Bit width Δt	1 ns	Bits of M sequence	$2^{7}-1$
Mean signal photoelectrons per bit $M_{s}$	1	Dead time	45 ns
Mean noise photoelectrons per bit	$2\times 10^{4}$		

**Table 2:** Main experimental parameters.

Photon detection efficiency of GM-APD	2%	Bits of M sequence	$2^{14}-1$
Bit width	4 ns	Dead time	40 ns
Noise Count	1Mcps	Time resolution of TCSPC module	64 ps
Wavelength	1064 nm		
